# Comparative Cytotoxic Activity of Hydroxytyrosol and Its Semisynthetic Lipophilic Derivatives in Prostate Cancer Cells

**DOI:** 10.3390/antiox10091348

**Published:** 2021-08-25

**Authors:** Antonio J. León-González, Prudencio Sáez-Martínez, Juan M. Jiménez-Vacas, Vicente Herrero-Aguayo, Antonio J. Montero-Hidalgo, Enrique Gómez-Gómez, Andrés Madrona, Justo P. Castaño, José L. Espartero, Manuel D. Gahete, Raúl M. Luque

**Affiliations:** 1 Maimonides Institute of Biomedical Research of Cordoba (IMIBIC), 14004 Cordoba, Spain; prudensama95@gmail.com (P.S.-M.); b12jivaj@uco.es (J.M.J.-V.); b22heagv@uco.es (V.H.-A.); b42mohia@uco.es (A.J.M.-H.); enriquegomezgomez@yahoo.es (E.G.-G.); justo@uco.es (J.P.C.); bc2gaorm@uco.es (M.D.G.); 2 Department of Cell Biology, Physiology and Immunology, University of Cordoba, 14014 Cordoba, Spain; 3 Reina Sofia University Hospital (HURS), 14004 Cordoba, Spain; 4 CIBER Physiopathology of Obesity and Nutrition (CIBERobn), 14004 Cordoba, Spain; 5 Department of Pharmacology, Faculty of Pharmacy, University of Seville, 41012 Seville, Spain; 6 Urology Service, HURS/IMIBIC, 14004 Cordoba, Spain; 7 Department of Organic and Pharmaceutical Chemistry, Faculty of Pharmacy, University of Seville, 41012 Seville, Spain; madrona@us.es (A.M.); jles@us.es (J.L.E.)

**Keywords:** anticancer, extra virgin olive oil, hydroxytyrosol, prostate cancer, semisynthetic derivatives

## Abstract

A high adherence to a Mediterranean diet has been related to numerous beneficial effects in human health, including a lower incidence and mortality of prostate cancer (PCa). Olive oil is an important source of phenolic bioactive compounds, mainly hydroxytyrosol (HT), of this diet. Because of the growing interest of this compound and its derivatives as a cancer chemopreventive agent, we aimed to compare the in vitro effect of HT isolated from olive mill wastewaters and five semisynthetic alkyl ether, ester, and nitro-derivatives against prostate cancer (PCa) cell lines. The effect in cell proliferation was determined in RWPE-1, LNCaP, 22Rv1, and PC-3 cells by resazurin assay, the effect in cell migration by wound healing assay, and tumorsphere and colony formation were evaluated. The changes in key signaling pathways involved in carcinogenesis were assessed by using a phosphorylation pathway profiling array and by Western blotting. Antiproliferative effects of HT and two lipophilic derivatives [hydroxytyrosyl acetate (HT-Ac)/ethyl hydroxytyrosyl ether (HT-Et)] were significantly higher in cancerous PC-3 and 22Rv1 cells than in non-malignant RWPE-1 cells. HT/HT-Ac/HT-Et significantly reduced migration capacity in RWPE-1 and PC-3 and prostatosphere size and colony formation in 22Rv1, whereas only HT-Ac and HT-Et reduced these functional parameters in PC-3. The cytotoxic effect in 22Rv1 cells was correlated with modifications in the phosphorylation pattern of key proteins, including ERK1/2 and AKT. Consistently, HT-Ac and HT-Et decreased p-AKT levels in PC-3. In sum, our results suggest that HT and its lipophilic derivatives could be considered as potential therapeutic tools in PCa.

## 1. Introduction

Dietary fruits and vegetables are the source of a wide variety of compounds with an interest in the treatment and prevention of cancer. In this sense, recent studies relate a high adherence to the Mediterranean diet with a lower incidence, aggressiveness, and mortality of prostate cancer (PCa) [1,2,3,4,5]. PCa is the second most diagnosed cancer type and the sixth cause of cancer death among men worldwide [6]. Frequently, PCa patients are initially diagnosed with a tumor with a low risk of progression (commonly referred to as non-significant PCa, non-SigPCa). However, up to 30–50% of patients progress to a clinically significant PCa (SigPCa) during the first 5–10 years, requiring active treatment [7]. Because of the usual slow course of the progression of this disease, there is a great interest in finding chemopreventive therapeutics with a low risk of side effects that could act in both the non-SigPCa and the lethal phenotype of PCa.

In this context, several studies have evaluated edible fruits and vegetables from the Mediterranean diet as the source of bioactive compounds with interest in the treatment and prevention of PCa [4,8]. Particularly, there is a growing concern in the role of bioactive compounds of extra virgin olive oil (EVOO), including phenolic alcohols [i.e., tyrosol, hydroxytyrosol (HT)] and their derivatives (i.e., oleocanthal and other secoiridoids) in different types of cancer [9]. Among them, HT exerted promising anticancer effects in previous in vitro and in vivo preclinical cancer models. This molecule can act both as antioxidant at low concentrations or prooxidant in long-term treatments and high concentrations, leading to apoptosis and cell cycle arrest of cancer cell lines, such as breast, liver, melanoma, and pancreas [10,11,12,13]. Numerous studies have explored the underlying anticancer mechanisms of hydroxytyrosol, including the inhibition of cAMP Response Element-Binding (CREB), p38, Extracellular-signal-Regulated Kinase 1/2 (ERK 1/2), c-Jun N-terminal Kinase (JNK), phosphatidylinositol 3-kinase (PI3K)/protein kinase B (AKT)/mechanistic target of rapamycin (mTOR), among other pathways [14,15,16]. Furthermore, it has been reported that olive phenolic alcohols and secoiridoids induce apoptosis and lead to cell cycle arrest in different PCa cell lines by the downregulation of pro-inflammatory and pro-angiogenic pathways and of the proto-oncogene receptor tyrosine kinase Met (c-Met) [17,18,19].

The olive mill wastewaters are a by-product of the olive oil industry, which were considered earlier as a great environmental problem, but are nowadays appreciated as an excellent source of HT and other biophenols with interest in agri-food, cosmetic and pharmaceutical industries [20]. In this line, Bassani et al. assessed the cancer chemopreventive activity of an HT-rich extract, obtained from olive mill wastewaters, named A009, in two human colon-carcinoma cell lines (HT-29, HCT-116) and in the murine colon-carcinoma cell line CT-26. This extract decreased cell growth, adhesion, migration, sprouting, and invasion in vitro, as well as reduced different signaling pathways related with angiogenesis and inflammation [21]. Moreover, A009 treatment was also active in vivo, as it significantly reduced the tumor weight in a murine xenograft model (CT-26 cells in syngeneic BalbC mice) [21].

Moreover, during the last years, new chemical derivatives of HT have been developed. Previous studies of our group reported the synthesis of ester and ether derivatives of HT and nitro-HT and revealed that some of these modifications could improve the absorption and pharmacological activities of HT, including antioxidant, anti-inflammatory, antiplatelet, neuroprotective, anti-angiogenic, and anticancer effects [22,23,24,25,26,27,28]. However, the effects of these HT derivatives in PCa are still poorly studied. 

**Figure 1:**
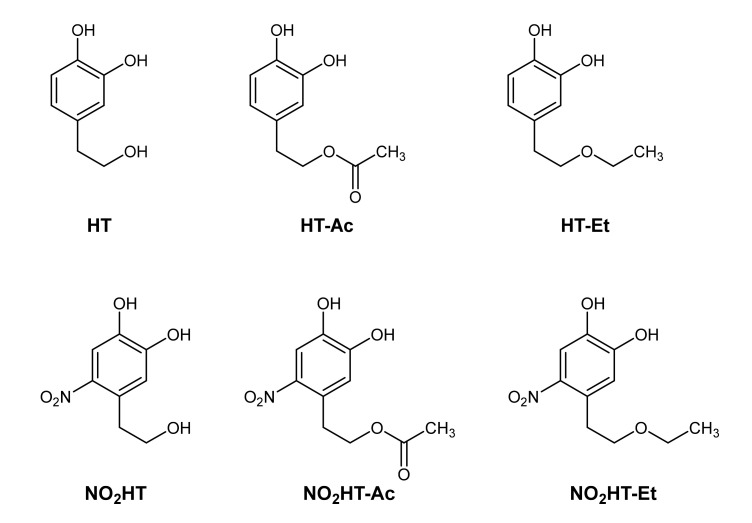
Chemical structures of the tested compounds. Hydroxytyrosol (HT) and derivatives: hydroxytyrosyl acetate (HT-Ac), ethyl hydroxytyrosyl ether (HT-Et), nitrohydroxytyrosol (NO_2_HT), nitrohydroxytyrosyl acetate (NO_2_HT-Ac), and ethyl nitrohydroxytyrosyl ether (NO_2_HT-Et).

This background led us to study the chemopreventive effect against PCa of HT, one of the main bioactive phytochemicals of olive oil, obtained from olive mill wastewaters, and five semisynthetic derivatives obtained by the introduction of ether, acetate, and/or nitro groups (Figure 1). These derivatives showed higher antioxidant capacity than HT in previous studies, and their increased lipophilicity improved their physic-chemical properties to be included in formulations of functional foods. We hypothesized that these derivatives could also improve the PCa chemopreventive effect of HT, as occurred in other pathologies. Therefore, the aim of this study was to determine and compare the in vitro anticancer effect and mechanism of action of HT and its semisynthetic derivatives in both non-malignant (RWPE-1) and cancerous (LNCaP, 22Rv1 and PC-3) prostate cell lines. This study would enable the evaluation of HT and its derivatives as potential novel phytochemicals in PCa chemoprevention, which could be used as part of functional foods or in the formulation of enriched EVOO.

## 2. Material and Methods

### 2.1. Chemicals 

Olive oil wastewaters were kindly supplied by the olive oil extraction factory “Oleícola El Tejar” in Córdoba, Spain. HT was extracted and purified by column chromatography (95% purity) as previously described [20]. The 5 selected HT derivatives, namely hydroxytyrosyl acetate (HT-Ac), ethyl hydroxytyrosyl ether (HT-Et), nitrohydroxytyrosol (NO_2_HT), nitrohydroxytyrosyl acetate (NO_2_HT-Ac), and ethyl nitrohydroxytyrosyl ether (NO_2_HT-Et), were synthesized as described elsewhere [24,27,29,30,31]. Chemical structures are showed in Figure 1. Stock solutions were prepared by dissolving compounds in dimethyl sulfoxide (DMSO), being its final concentration < 0.1% (*v/v*). DMSO dissolved in culture medium was used as a control vehicle.

### 2.2. Cell Culture 

Cell lines derived from normal prostate (RWPE-1) and from androgen-dependent (LNCaP), sensitive (22Rv1) and non-dependent (PC-3) PCa were obtained from the American Type Culture Collection (Manassas, VA, USA) and cultured in a humidified incubator with 5% CO_2_ at 37 °C according to manufacturer instructions, as previously described [32,33,34]. An analysis of short tandem repeat sequences (STRs) was performed to validate these cell lines by using GenePrint 10 System (Promega, Barcelona, Spain), and the absence of mycoplasma was confirmed by PCR as previously reported [35].

### 2.3. Cell Proliferation 

Cell proliferation was assessed by resazurin reagent (Canvax Biotech, Cordoba, Spain), as previously reported [32,35]. Briefly, 3000 to 5000 cells were seeded per well in 96-well plates, serum-starved overnight, and then exposed to different concentrations (0–300 µM) of compounds for 48 h. Cell proliferation was assessed before and after 48 h of treatment by measuring the fluorescence (560 nm excitation and 590 nm emission) after 3 h incubation with 10% resazurin by using a FlexStation III system (Molecular Devices, Sunnyvale, CA, USA). Results were expressed as a percentage of proliferation referred to as control (DMSO vehicle-treated). At least 3 experiments with 4 replicates of each condition were performed. Paclitaxel, a cytotoxic taxane commonly used in PCa chemotherapy, was used as an internal positive control.

### 2.4. Cell Migration Assay

Cell migration was evaluated by wound healing assay as previously described [35,36]. Briefly, 50,000 cells were seeded in 96-well plates and grown until confluence. Wounds were made with IncuCyte WoundMaker (Essen BioScience, Ann Arbor, MI, USA) according to the manufacturer’s instructions. Wells were then washed with PBS and cells were treated overnight with 0–100 µM of HT or derivatives in serum-free medium. Images of the scratch were taken just after wounding (0 h) and at the end of the treatment. Wound healing was calculated as the ratio between the scratch areas at these 2 timepoints, measured with ImageJ software (National Institutes of Health, Bethesda, MD, USA). Results were expressed as the percentage of migration rate referred to as control. At least 3 experiments with 3 replicates of each condition were performed. This experiment was performed with RWPE-1 and PC-3 cell lines but not with 22Rv1 or LNCaP cells due to their lower migration capacity.

### 2.5. Clonogenic Assay

To assess the clonogenic capacity of PC-3 and 22Rv1 PCa cells, 2000 cells were seeded into 6-well plates, treated with 0, 10, or 20 µM of HT or its derivatives and incubated for 10 days. The medium was then removed, and the colonies were washed with PBS and stained with crystal violet solution (6% glutaraldehyde, 0.5% crystal violet) for 30 min, rinsed and air-dried. At least 3 experiments with 2 replicates of each condition were performed. The number of colonies was counted by using ImageJ software (National Institutes of Health, Bethesda, MD, USA). Results were expressed as a percentage of the number of colonies referred to as control.

### 2.6. Prostatosphere Formation

Prostatosphere formation assay was carried out in representative cell models of advanced PCa (22Rv1 and PC-3), as previously reported [33,37]. Briefly, 2000 cells/well were seeded in Corning Costar 24-well ultra-low attachment plates (Merck, Madrid, Spain) with DMEM F-12 medium supplemented with 20 ng/mL EGF (Sigma-Aldrich, Madrid, Spain). Treatments were added while plating the cells and refreshed every 3 days. The area of prostatospheres was determined after 14 days of incubation with ImageJ software. At least 3 experiments with 2 replicates of each condition were performed. Results were expressed as percentage of prostatosphere area referred to as control.

### 2.7. Phosphorilation Array

Protein extracts of 22Rv1 cells were collected in lysis buffer from 6-well plates after 24 h treatment with DMSO dissolved in culture medium (control), HT, HT-Ac, or HT-Et 10 µM. A phosphorylation pathway profiling array was performed by using the Human Phosphorylation Pathway Profiling Array C55 kit #AAH-PPP-1–8, following the manufacturer’s instructions (Raybiotech, Inc., Peachtree Corners, GA, USA). Briefly, membranes for the semi-quantitative detection of 55 phosphorylated human proteins belonging to the MAPK, AKT, JAK/STAT, NFκB, and TGFβ signaling pathways were incubated for 30 min with blocking buffer at 25 °C and then incubated overnight at 4 °C with 1 mL of a 4-fold dilution of 22Rv1 cell lysates (*n* = 4; pooled). After washing, the membranes were incubated with a detection antibody cocktail at 25 °C for 2 h and, next with horseradish peroxidase (HRP)-labeled anti-rabbit secondary antibody at 25 °C for an additional 2 h. The signals were collected after adding ECL reagent by a chemiluminescence detection system BioRad Universal Hood II (BioRad Laboratories, Hercules, CA, USA). Densitometric analysis of the array spots was quantified by using ImageJ software. Positive control spots were used as a normalizing factor. Results were expressed as log2 of the fold change of the signal of each protein, compared with the signal of control. A log2 Fold Change of 0.2 was considered as a threshold. Clustering was performed by using String v11.0 (https://string-db.org, accessed on 1 July 2021). 

### 2.8. Western Blot Analysis

Proteins from whole-cell lysates were extracted, separated on 10% SDS-polyacrylamide gels, and transferred onto nitrocellulose membranes, as previously described [35,36]. Membranes were then probed overnight at 4 °C with an appropriate primary antibody anti-phospho-AKT, phospho-ERK, AKT, or ERK (Cell-Signaling Technology, Danvers, MA, USA). Membranes were thereafter incubated for 1 h with the corresponding horseradish peroxidase-linked secondary antibody (HRP-conjugated goat anti-rabbit IgG, Cell-Signaling). Immunoreactive bands were detected using ECL chemiluminescence substrate solution (GE Healthcare Europe GmbH, Madrid, Spain) in an enhanced chemiluminescence detection system (GE Healthcare, Madrid, Spain). Observed bands were quantified by using ImageJ software, and results were expressed as a percentage of control.

### 2.9. Statistical Analysis 

All the experiments were performed in at least 3 independent experiments (*n* ≥ 3). The half inhibitory concentration (IC_50_) values were calculated using the nonlinear regression analysis of cell proliferation. Statistical differences between 2 variables were calculated by unpaired parametric t-test and nonparametric Mann–Whitney U test, according to normality, assessed by Kolmogorov–Smirnov test. For differences among three variables, One-Way ANOVA analysis was performed. Statistical significance was considered when *p* < 0.05. All the analyses were assessed using GraphPad Prism 8 (GraphPad Software, La Jolla, CA, USA). 

## 3. Results and Discussion

### 3.1. Hydroxytyrosol and Five Semisynthetic Derivatives Exert a Concentration-Dependent Effect in the Proliferation of Prostate Cells

**Figure 2:**
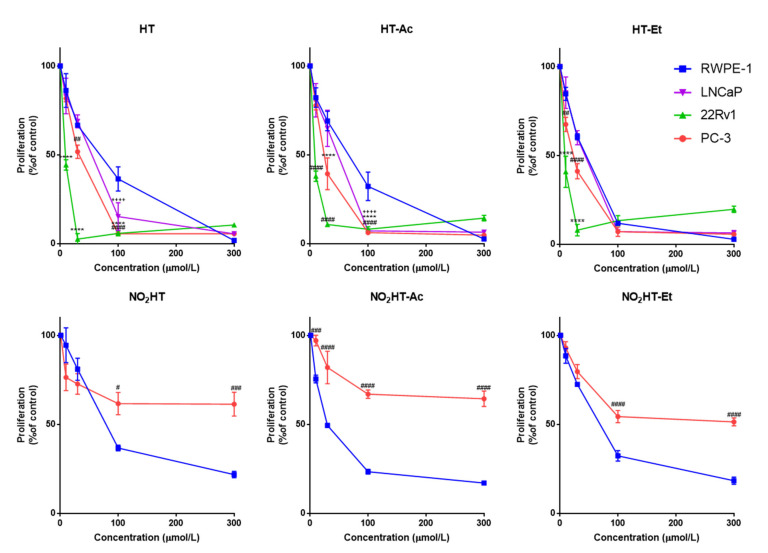
Concentration-response curves on cell proliferation of HT and derivatives (48 h treatment) in normal RWPE-1, and in cancer LNCaP, 22Rv1, and PC-3 cell lines. Data are expressed as percent of control proliferation (set at 100%) and represent the mean ± SEM (4 experiments) vs. each compound concentration (µmol/L). Symbols (^+^ LNCaP, * 22Rv1, ^#^ PC-3) indicate statistically significant differences with the corresponding RWPE-1 value: ^++++^ *p* < 0.0001, * *p* < 0.05, ** *p* < 0.01, *** *p* < 0.001, **** *p* < 0.0001, ^##^ *p* < 0.01, ^###^ *p* < 0.001, ^####^ *p* < 0.0001. Abbreviations: HT, Hydroxytyrosol; HT-Ac, hydroxytyrosyl acetate; HT-Et, ethyl hydroxytyrosyl ether; NO_2_HT, nitrohydroxytyrosol; NO_2_HT-Ac, nitrohydroxytyrosyl acetate; NO_2_HT-Et, ethyl nitrohydroxytyrosyl ether.

To compare the antiproliferative effect of five HT derivatives with the parent compound, the proliferation rate of the non-tumor prostate epithelial cell line, RWPE-1, and the tumor cell line PC-3 was measured after 48 h of incubation with these compounds (0, 10, 30, 100, and 300 µM; Figure 2). Specifically, the proliferation capacity of both cell lines decreased after treatment with all the tested compounds in a range of concentrations of 30 to 300 µM (Figure 2). Native HT and two of its derivatives, HT-Ac and HT-Et, had a selective antiproliferative effect against cancer cells, as it was significantly higher in tumor PC-3 cells rather than in RWPE-1 at specific concentrations (HT exerted a selective effect at 100 µM, whereas HT-Ac was selective at 30 and 100 µM and HT-Et was at 10 and 30 µM, respectively). This result agrees with previous studies, where HT and alkyl ether derivatives, including HT-Et induced a higher cytotoxic effect in lung or prostate cancer cells than in the corresponding normal cells [19,28].

In contrast, the nitro-derivatives were more cytotoxic in the non-malignant cells. This result indicates that the introduction of a nitro group reduces the selective anticancer effect of HT. Moreover, as shown by López-Jiménez et al., the nitro-containing HT derivatives were much weaker antiangiogenic compounds, whereas HT-Ac and HT-Et exerted a greater inhibition of the formation of tubule-like structures by endothelial cells in Matrigel [23]. Consequently, HT, HT-Ac and HT-Et, but not the nitro-derivatives, were selected for further experiments in the present study.

To corroborate the antiproliferative activity of HT and its derivatives, HT-Ac and HT-Et, in PCa, they were also tested in the androgen-dependent cell line LNCaP and in the androgen-sensitive 22Rv1 cell line. As occurred in PC-3, a concentration-dependent significant antiproliferative effect of these compounds was also observed in both LNCaP and 22Rv1 cells (Figure 2). Given that AR-positive but also AR-negative cells (i.e., PC-3) were sensitive to HT, HT-Ac, and HT-Et, we hypothesized that the mechanism of action of these treatments, at least at the tested concentrations, were independent of AR signaling.

The half inhibitory concentrations (IC_50_, µM) of the selected compounds in the four cell lines are presented in Table 1. In general, the derivatives exerted a slightly higher cytotoxic effect against the tumor cell lines than HT. This is consistent with previous studies showing that the chemical modification of the parent compound HT, by introducing acetate or ethyl ether groups, increases the cytotoxic effect in cancer cells, including human Caco-2 colon adenocarcinoma and A549 lung cancer cells [27,28,38]. Moreover, it has been described that the semisynthetic HT-Et derivative showed enhanced chemical stability compared to HT and HT-Ac [39].

Specifically, the three compounds showed lower IC_50_ values in the cancer cell lines than in the non-tumor RWPE-1 cells. The cytotoxic effect of these compounds was higher in the 22Rv1 cell line, where they exerted a selective antiproliferative effect at the low micromolar range, rather than in PC-3 and LNCaP cells. This is in accordance with previous studies, showing that after HT treatment for 24 h, the cytotoxic effect depended on the Phosphate Tensin homolog (*PTEN*) status of the cell line [40]. *PTEN* is considered a tumor suppressor gene that is commonly mutated or deleted in PCa patients, leading to a functional loss, which is related with higher aggressiveness of the disease and a poorer clinical outcome [41]. In this sense, we observed that *PTEN*-null LNCaP and PC-3 cells (−/−) were most resistant, whereas the 22Rv1 cells (+/+) were the most sensitive. This result suggests that the cytotoxic effect of HT, HT-Ac, and HT-Et in 22Rv1 cells is conditioned, at least in part, by activation of PTEN, which, in turn, inhibits the pro-proliferative effect of the PI3K–AKT–mTOR pathway. This is in accordance with an early study comparing the alteration of different signaling pathways in four PCa cell lines (LNCaP, 22Rv1, DU-145, and PC-3) after treatment with cisplatin [42]. The cell lines with functional PTEN, i.e., DU-145 and 22Rv1 were the most sensitive to the apoptosis induction with this drug, suggesting that the PTEN status was more relevant than the androgen receptor (AR) status to determine the response of certain cytotoxic pro-apoptotic drugs. 

Moreover, this PTEN-dependent effect of HT has been also related to its prooxidant activity, as similar results were obtained after treatment with HT or with the prooxidant agent *N,N,N′,N′*-tetrakis (2-pyridylmethyl) ethylenediamine (TPEN) in 22Rv1, DU-145 and PC-3 PCa cell lines [40], suggesting that both HT and its semisynthetic derivatives also exert a prooxidant effect. The prooxidant effect of HT was corroborated by Luo et al. as they evidenced that the in vitro cytotoxic effect of this phenolic compound of the olive oil depended on the generation of superoxide in PC-3 cells leading to mitochondrial dysfunction and apoptosis [43].

These observations, together with previous studies showing an increase of reactive oxygen species generation in PC-3 cells treated with HT, which was correlated with the induction of apoptosis [43], pointed out that the cytotoxic effect of HT and its derivatives in PCa cells could be mediated by a prooxidant redox mechanism. 

The IC_50_ values obtained for HT and the selected derivatives after 48 h of treatment are in the low micromolar range (7 to 50 µM). Previous studies estimated the daily consumption of olive oil phenolic compounds in 5.4–9.0 mg, approximately 35–58 µmol of HT-equivalents per day [38]. Moreover, Covas et al. measured plasma levels of nearly 20 µM HT after the ingestion of 40 mL of olive oil. Because higher plasma concentrations are hardly affordable in a standard diet, the development of functional foods such as a polyphenol-enriched oils or phytomedicines, including these lipophilic derivatives, constitutes a possible strategy to intake of these chemopreventive phytochemicals [44].

### 3.2. HT, HT-Ac, and HT-Et Decrease Migration Capacity of Prostate Cells in A Concentration-Dependent Manner

**Figure 3:**
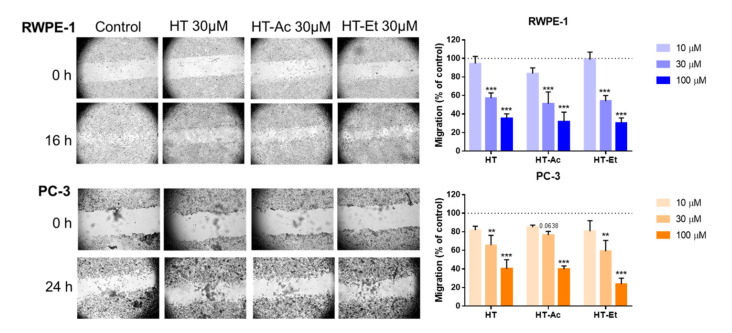
Cell migration rate of RWPE-1 (16 h), and PC-3 (24 h) treated with 0–100 µM of HT, HT-Ac, and HT-Et. The micrographs (magnification 40×) illustrate representative captures of the RWPE-1 and PC-3 cells before and after treatment with vehicle (DMSO, control) or 30 µM of HT and derivatives. Data are expressed as migration (percent of control, set at 100%) and represent the mean ± SEM (4 experiments). Asterisks indicate significant differences to DMSO control, set at 100% and represented as a dashed line: ** *p* < 0.01, *** *p* < 0.001. Abbreviations: HT, Hydroxytyrosol; HT-Ac, hydroxytyrosyl acetate; HT-Et, ethyl hydroxytyrosyl ether.

To further compare the effect of HT and the selected derivatives against PCa, the migration of RWPE-1 and PC-3 cells was assessed after incubation with 0–100 µM of these compounds. Treatment with HT, HT-Ac, and HT-Et significantly reduced the migration rate of RWPE-1 and PC-3 cells in a concentration-dependent manner (Figure 3). Previous studies reported that different olive biophenols, including HT and oleuropein, inhibit migration and invasion of other cancer cell types, such as triple-negative breast cancer (TNBC) MDA-MB-231 cells by stimulating autophagy [45]. Similarly, HT also inhibited the migration capacity and invasion of MDA-MB-231 and other TNBC cell lines (i.e., SUM159PT and BT549) through a reconstituted basement membrane in the Boyden chamber assay, via the alteration of epithelial-to-mesenchymal transition (EMT) and embryonic signaling pathways [12].

Moreover, 24 h pre-treatment of PC-3, DU-145, and LNCaP cells with an HT-rich extract from olive mill wastewaters (A009) significantly reduced the cell adhesion, migration, and invasion of the PCa cell lines [46]. Nevertheless, to the best of our knowledge, this is the first report showing the inhibition of migration capacity in PCa cells mediated by the HT derivatives HT-Ac and HT-Et.

Regarding RWPE-1 cells, although these are non-malignant prostate cells, they have a considerable ability of migration and invasion. In this sense, the decrease of the migration rate after treatment with HT or its derivatives is in consonance with the effect observed after treating RWPE-1 cells with other PCa chemopreventive agents, such as finasteride [47]. 

### 3.3. Effect of HT, HT-Ac, and HT-Et in Prostate Cancer Stem Cells 

**Figure 4:**
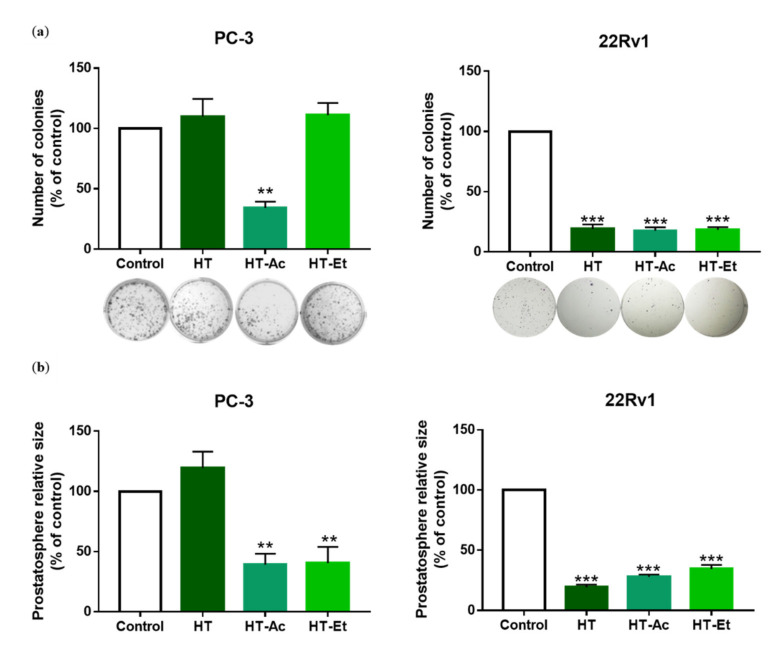
(**a**) Effect of HT, HT-Ac, and HT-Et treatment of PC-3 (20 µM, 10 days) and 22Rv1 (10 µM, 14 days) cells in the clonogenic assay. Data are expressed as number of colonies (percent of control, set at 100%) and represent the mean ± SEM (4 experiments). Asterisks indicate significant differences compared to the control value: ** *p* < 0.01, *** *p* < 0.001; (**b**) Effect of HT, HT-Ac, and HT-Et treatment of PC-3 (20 µM, 14 days) and 22Rv1 (10 µM, 14 days) in the size of prostatospheres. Data represent the mean area ± SEM (4 experiments). Asterisks indicate significant differences to the control value: ** *p* < 0.01, *** *p* < 0.001. Abbreviations: HT, Hydroxytyrosol; HT-Ac, hydroxytyrosyl acetate; HT-Et, ethyl hydroxytyrosyl ether.

PCa stem cells play a key role in the initiation of PCa and the development of metastasis [48]. The functional effect of the treatment with 10 µM (for 22Rv1) or 20 µM (for PC-3) of the selected compounds was studied by prostatosphere and clonogenic assays (Figure 4). These concentrations were selected in accordance with IC_50_ values obtained in proliferation assay and are similar to plasma concentrations of HT observed in volunteers after ingestion of EVOO [49]. Treatment with each of the three compounds significantly reduced the prostatosphere size and the number of colonies in 22Rv1 cells. It should be considered that the antiproliferative effect of HT and its derivatives could negatively affect the formation of colonies and prostatospheres. However, previous studies suggest that HT was able to reduce cancer stem cell markers in other types of cancer, such as CD44 in breast cancer cells [12]. According to Cruz-Lozano et al., this effect could be mediated by a reduction of epithelial to mesenchymal transition (EMT) related markers and the Wnt/β-catenin signaling pathway, as they observed a decrease of ALDH^+^ (aldehyde dehydrogenase) and CD44^+^/CD24^−/low^ subpopulations, and of the number of tumorspheres of breast cancer stem cells after treatment with HT accompanied by a modulation of these pathways [12].

In PC-3 cells, HT-Ac and HT-Et, but not HT, reduced the prostatosphere size, while only treatment with HT-Ac significantly diminished the formation of cell colonies, lowering the number of colonies to a 40% of control. These results suggest that HT derivatives, especially HT-Ac, could improve the anticancer effect of HT against cancer stemness of certain PCa cells that, as PC-3, are deleted in *PTEN*, a tumor suppressor gene. In fact, PTEN loss has been related with an increase of cancer stem-like populations in PCa [48,50], which, in turn, has been correlated with the activation of Wnt/β-catenin activation [51].

### 3.4. HT, HT-Ac, and HT-Et, Modulate Key Signaling Pathways in 22Rv1 Cells

**Figure 5:**
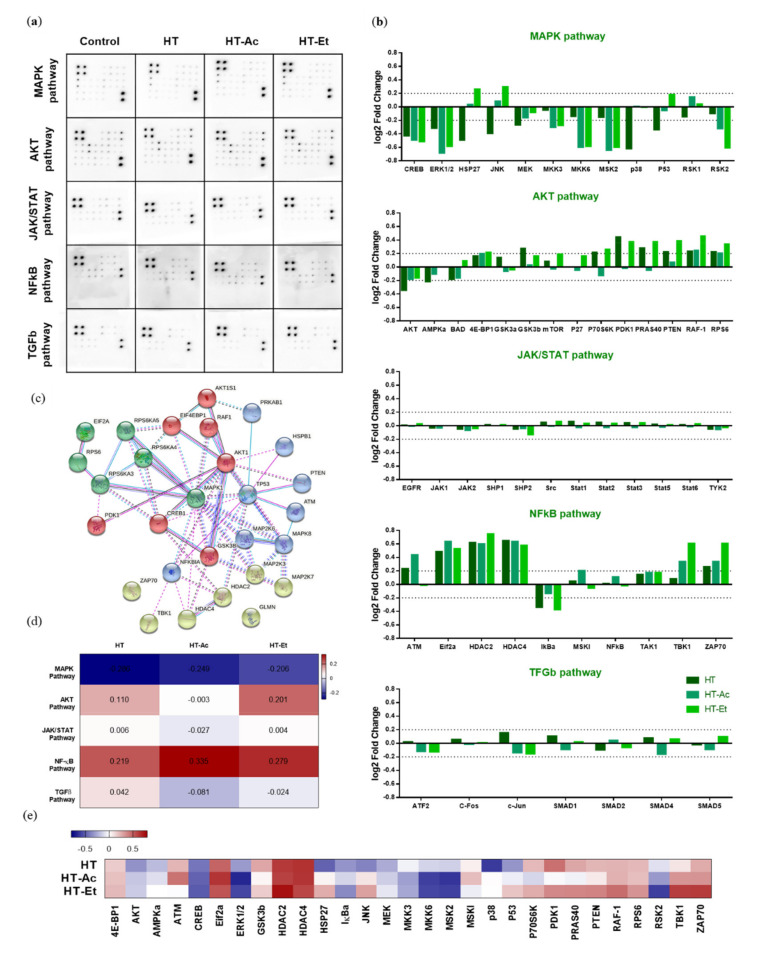
Effect of HT, HT-Ac, and HT-Et treatment (10 µM, 24 h) in the phosphorylation of key proteins in cancer-related pathways in 22Rv1 cells. (**a**) Membranes showing the spots quantified to study the phosphorylation level of 55 proteins in the phosphoprotein array (5 oncogenic pathways; 55 phosphorylated proteins) in response to 24 h treatment HT, HT-Ac, and HT-Et (*n* = 4; pooled). (**b**) log2 Fold Change of phosphorylation protein level in comparison with the control (DMSO) condition (threshold: log2 Fold Change = 0.2). (**c**) The significantly altered phosphorylated proteins were analyzed using the STRING database. (**d**) Heatmap showing the log2 Fold Change mean corresponding to each pathway. (**e**) Heatmap showing the log2 Fold Change of selected altered proteins. Abbreviations: HT, Hydroxytyrosol; HT-Ac, hydroxytyrosyl acetate; HT-Et, ethyl hydroxytyrosyl ether.

To further explore the underlying mechanism of the cytotoxic activity of HT and its derivatives in 22Rv1 PCa cells, we performed a semi-quantitative analysis of the phosphorylation status of 55 proteins, encompassed in five key pathways that are involved in carcinogenesis, i.e., MAPK, AKT, JAK/STAT, NFκB, and TGFβ (Figure 5). 

The Ras/mitogen-activated protein kinase (MAPK) family represents a pivotal pathway in PCa evolution, as the phosphorylation of extracellular signal-regulated kinases (ERK1/2, aka MAPK1/2) activates a cascade of signals that ultimately promotes the translocation of transcription factors to the nucleus, leading to the regulation of genes encoding proteins with crucial roles in proliferation, such as cyclin D1 and other processes, including angiogenesis, metastasis, and chemo-resistance [52]. 

Aberrant activation of PI3K/AKT and MEK/ERK pathways is related to the initiation and progression of different types of cancer, including PCa [53]. The treatment with either HT or its derivatives clearly decreased the phosphorylation of ERK1 (T202/Y204) and ERK2 (Y185/Y187) in 22Rv1 cells. Similarly, the phosphorylation of other members of the same pathway, such as MKK3 (S189) and MKK6 (S207), as well as AKT (S473), was also reduced. These phosphorylation inhibitions were consistent after the treatment with the three compounds and constituted the center of the clustered pathway (Figure 5c). A previous study by Kharaziha et al. reported that 22Rv1 cells exhibit constitutive activation of ERK1/2 and that its inhibition with the multi-tyrosine kinase inhibitor sorafenib induced cell death via activation of the pro-apoptotic protein Bad [54]. Likewise, the prooxidant agent 1-(3,5-dimethylphenyl)-6-methyl-1H-pyrazolo[4,3-c]pyridin-4 (5H)-one (DPMPP) exerted a cytotoxic effect in 22Rv1 cells by causing cell cycle arrest in S phase and apoptosis accompanied with an increase in the reactive oxygen species production and with an inhibition of the PI3K/AKT/ERK phosphorylation [55]. 

The treatment of 22Rv1 cells with HT, HT-Ac, or HT-Et significantly reduced the phosphorylation levels of cAMP response element-binding (CREB) protein. This transcription factor directly binds to ABCG2 promoter to regulate the expression of ABCG2, a well-known marker for PCa stem cells [56,57]. The phosphorylation of CREB was associated with gastric tumor chemo-resistance driven by gastric cancer stem cell-like via increased levels of ABCG2 [58]. Moreover, p-CREB is the target of other markers of cancer stem-like cells and epithelial–mesenchymal transition, such as CD44 [59]. Concretely, it has been described that thyroid cancer cell proliferation was stimulated after ERK-mediated phosphorylation of CREB and the stabilization of p-CREB through CD44 [59]. These observations suggest that the decrease on CREB phosphorylation could mediate the reduction of the cancer stem-like properties of 22Rv1 cells.

Another transcription factor involved in PCa cell stemness and inflammatory processes is NFκB [60]. The 24 h treatment with 10 µM of the selected compounds was unable to reduce the phosphorylation of NFκB, although previous studies showed a decrease in p-NFκB levels after treating PC-3, DU-145, and LNCaP cells with the HT-rich extract A009. This difference could be explained by the differences on the HT concentration or the presence of other bioactive compounds in this A009 olive mill wastewater extract [46]. Similarly, another study also reported the inhibition of the NFκB pathway in LNCaP cells after treatment with HT at a 10-fold higher concentration (100 µM) [19]. Nevertheless, we observed a decrease in the phosphorylation levels of IκBα, which inhibits its degradation and, in turn, impedes the translocation of NFκB from the cytoplasm to the nucleus. Thus, the treatment of 22Rv1 with HT/HT-Ac/HT-Et 10 µM could downregulate this pathway by inhibiting the nuclear translocation of NFκB.

JNK is also known as stress-activated MAP kinase (SAPK), and it has been correlated with the regulation of numerous processes in PCa, including apoptosis, proliferation, tumorigenesis, and inflammation [61]. The HT-Et treatment increased the phosphorylation of C-Jun N-terminal kinase (JNK), whereas HT decreased it. The apparently contradictory effect of these compounds can be explained as these signaling pathways are complex and can be modulated by numerous circumstances. In fact, previous studies have described that either the inhibition of JNK phosphorylation by naringenin or the increase of the p-JNK (Thr183/Tyr185) levels in LNCaP by green tea polyphenols were able to exert a cytotoxic effect in cancer cells [62,63].

### 3.5. HT-Ac and HT-Et, but Not HT, Reduce the Activation of AKT in PC-3 Cells

After observation of the central role of ERK and AKT phosphorylation in the cytotoxic effect of HT, HT-Ac and HT-Et treatment in 22Rv1 cells, the effect in AKT and ERK activation was assessed by Western blot in PC-3 cells treated with 20 µM of the selected compounds for 24 h. 

**Figure 6:**
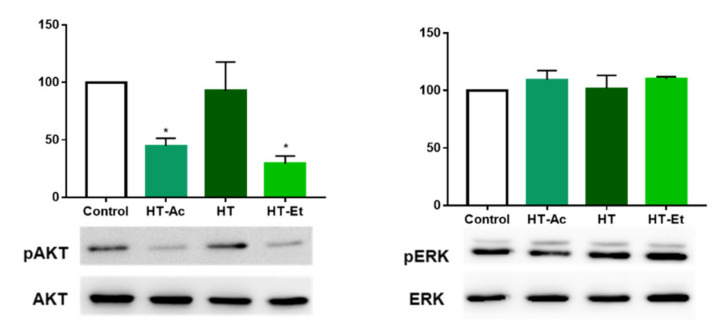
Effect of HT-Ac, HT, and HT-Et treatment (20 µM, 24 h) in the phosphorylation of AKT and ERK in PC-3. Data are calculated as the ratio between pAKT/AKT and pERK/ERK levels, respectively (expressed as percent of control, set at 100%), and represent the mean ± SEM (4 experiments). Asterisks indicate significant differences compared to the control value: * *p* < 0.05. Abbreviations: HT, Hydroxytyrosol; HT-Ac, hydroxytyrosyl acetate; HT-Et, ethyl hydroxytyrosyl ether.

HT-Ac and HT-Et, but not HT, were able to reduce the phosphorylation of AKT at the selected concentration in PC-3 (Figure 6). As previously mentioned, PC-3 cells are deficient in *PTEN*, which has been related with elevated levels of p-AKT. We have observed that the reduction of the activation of AKT after HT-Ac and HT-Et was accompanied by the reduction of the prostatosphere size. This is in accordance with previous studies showing that AKT inhibition was able to avoid the stem cell-like properties of DU145 and 22Rv1 PCa cells depleted in *PTEN*, such as prostatosphere formation and CD44^+^/CD133^+^ and ALDH-positive cell populations [50]. These results suggest that the derivatives improve the anticancer effect of HT against PC-3 cells at the studied concentration, avoiding the cancer stemness of PC-3 cells by downregulation of the AKT pathway.

In contrast, ERK phosphorylation was not affected by the treatment of PC-3 cells with any of the assayed phenolic compounds. This is in accordance with Kharaziha et al., which observed that the p-ERK levels were not as much as relevant in the effect of the anticancer compound sorafenib in PC-3 cells as it was in 22Rv1cells [54]. 

## 4. Conclusions

Altogether, our data demonstrate that HT, HT-Ac, and HT-Et decrease the proliferation of 22Rv1 and the proliferation and migration rate of PC-3 PCa cells in a concentration-dependent manner. The lipophilic derivatives not only maintained the anticancer effect of the parent compound HT against PC-3 PCa cells but also improved different anticancer effects, such as the reduction of the prostatosphere formation and the AKT phosphorylation in PC-3 cells. These results, together with earlier studies showing an increase in the antioxidant and antiangiogenic capacity of HT-Ac and HT-Et, suggest that, in addition to HT, these derivatives could also be considered as potential therapeutic tools in PCa as well as to be incorporated into functional foods. Further studies are necessary to corroborate the pharmacokinetics and the in vivo effectivity of these compounds in preclinical models. Specifically, the cytotoxic effect of these compounds could be tested in vivo in 22Rv1 xenografts implanted in nude mice [64], as a model of PCa with hyperactivation of p-ERK, or in LNCaP or PC-3 xenografted mice, as these cells are *PTEN*-null and the AKT pathway is highly activated [65]. Otherwise, the *Pten* gene-conditional knockout mouse carcinogenesis model is considered highly desirable to study the potential chemopreventive activity of these compounds in PCa [66]. Alternatively, the Transgenic Adenocarcinoma of the Mouse Prostate (TRAMP) also mimics the natural progression of PCa, developing lesions that range from preneoplastic to metastasis [67]. These further studies with animal models would help to deepen on the effectivity of these compounds and their underlying mechanism of action.

## Figures and Tables

**Table 1:** Half inhibitory concentrations (IC_50_) of proliferation rates of RWPE-1, LNCaP, 22Rv1, and PC-3 cell lines after 48 h of treatment with HT and selected derivatives. Data are expressed as the mean of IC_50_ (µM) ± SEM (4 experiments).

Compound	RWPE-1	LNCaP	22Rv1	PC-3
**HT**	52.20 ± 4.19	41.17 ± 2.79 (*p*, 0.0996)	9.32 ± 0.50 *** (*p* < 0.0001)	28.88 ± 2.25 ** (*p*, 0.0010)
**HT-Ac**	54.18 ± 11.76	35.04 ± 3.68 ** (*p*, 0.0072)	7.65 ± 0.50 *** (*p* < 0.0001)	23.40 ± 3.20 **** (*p*, 0.0001)
**HT-Et**	35.25 ± 1.58	33.73 ± 1.51 (*p*, 0.9882)	9.18 ± 1.48 *** (*p*, 0.0003)	20.30 ± 3.09 * (*p*, 0.0419)

Asterisks indicate significant differences to the respective RWPE-1 value: * *p* < 0.05, ** *p* < 0.01, *** *p* < 0.001, **** *p* < 0.0001. Abbreviations: HT, Hydroxytyrosol; HT-Ac, hydroxytyrosyl acetate; HT-Et, ethyl hydroxytyrosyl ether.

## Data Availability

Data is contained within the article.
